# Increased Plasma Levels of the TH2 chemokine CCL18 associated with low CD4+ T cell counts in HIV-1-infected Patients with a Suppressed Viral Load

**DOI:** 10.1038/s41598-019-41588-1

**Published:** 2019-04-12

**Authors:** Prashant Malhotra, Patrick Haslett, Barbara Sherry, David H. Shepp, Paul Barber, Jonathan Abshier, Upal Roy, Helena Schmidtmayerova

**Affiliations:** 1 Division of Infectious Disease, Northwell Health, Manhasset, NY USA; 2 Department of Microbiology and Immunology, University of Miami, Miller School of Medicine, Miami, FL USA; 3 Center for Immunology & Inflammation, The Feinstein Institute for Medical Research, Manhasset, NY USA; 4 Donald and Barbara Zucker School of Medicine at Hofstra/Northwell, Hempstead, NY USA; 5 Florida International University, Miami, FL USA; 6 Department of Health and Biomedical Sciences, University of Texas Rio Grande Valley (UTRGV), Brownsville, TX USA; 7 Department of Biological Sciences, Florida International University, Miami, FL USA

## Abstract

The chemokine (C-C motif) chemokine ligand 18 (CCL18) is a structural homolog of CCL3 primarily produced by monocyte-derived cells with an M2 phenotype. Elevated levels of CCL18 have been observed in several diseases associated with malignancies and chronic inflammation. The role of CCL18 in Human Immunodeficiency Virus (HIV-1) infection remains unknown. We analyzed expression levels of T helper cell-mediated (TH2) chemokines CCL18, CCL17, and CCL22 by ELISA in plasma collected from HIV-1-infected and healthy donors. In HIV-1-infected individuals, plasma viral loads were monitored by NucliSense HIV-1 QT assay and T cell counts and expression of the activation marker CD38 were determined by flow cytometry. Our data showed a significant increase in plasma levels of CCL18 in HIV-1-infected individuals compared to uninfected controls (p < 0.001) and a significant correlation between CCL18 levels and viral load in untreated patients. No significant difference of CCL18 levels was detected among the HIV-1-infected patients treated with combined antiretroviral therapy (cART) and HIV-1-untreated patients.CCL18 values are negatively correlated with CD4+CD38+ cell numbers and total CD4+ T cell counts in patients with a suppressed viral load. Notably, plasma levels of the TH2 chemokines CCL17 and CCL22 are also elevated during HIV-1 infection. However, no correlation of CCL17 and CCL22 production with CD4+ T cell counts was detected. Presented data shows that the chemokines, CCL17, CCL18, and CCL22 are increased during HIV-1 infection. However, only increased levels of CCL18, a marker of M2 macrophages, correlate with low CD4+ T cell counts in patients with suppressed viral load, raising the possibility that CCL18 and/or CCL18-producing cells may interfere with their reconstitution in HIV-1-infected patients on cART.

## Introduction

HIV-1 infection is characterized by a complex immune disorder that presents itself as a decrease in CD4+ T cells, chronic immune activation, and imbalanced cytokine and chemokine production and macrophage dysfunction^1^. Macrophages are the second major target of HIV-1 infection^2,3^. Previous *in vitro* studies addressing the consequences of M1 and M2 macrophage polarization on HIV-1 infection reported that R5 HIV-1 infection is suppressed in M1 macrophages, whereas M2 macrophages exhibited a higher level of infection, as well as higher levels of HIV-1 DNA synthesis^3–5^.

Chemokines constitute a large family of chemotactic cytokines that direct migration of immune cells through the body under physiological and immunopathological conditions^6,7^. Chemokines are broadly divided into two groups, constitutive and inflammatory. Constitutively expressed chemokines have housekeeping functions and are involved in the homing of immune cells. Inflammatory chemokines, which are induced or up-regulated by inflammation, play a role in attracting immune cells to sites of infection and inflammation^7^. In addition, chemokines and chemokine receptors represent an important component of T helper cell (TH1 and TH2) mediated immune responses^8,9^.

CCL18, a structural homolog of CCL3 belongs to a subset of Beta-chemokines associated with TH2 responses^10^. Biologically active CCL18 is constitutively expressed in plasma of healthy individuals^11^ and levels are further increased under certain pathological conditions. Relatively high CCL18 levels have been detected in lung and in lymphoid organs of healthy individuals^12^. Cellular sources of CCL18 are mainly restricted to monocytes/macrophages and dendritic cells. Monocyte/macrophages constitutively express low levels of CCL18 and its expression is up-regulated by the TH2 cytokines IL-4, IL-10, and IL-13^13,14^. Besides TH2 cytokines, mechanisms that trigger generation of M2 macrophages, such as blockade of the co-stimulatory pathway B7/CD28, lead to increased expression of CCL18^15^. Immature Dendritic cells or DCs produce relatively high levels of CCL18, while maturation down-regulates CCL18 production by DCs^16^. CCL18 triggers biological responses in target cells and induces migration of T cells^13^, B cells^17^, and macrophages^18^ via CCL18 receptor CCR8^19^.

Increased levels of CCL18 have been detected in various pathological conditions^19,20^. Genetic variation in the CCL18-CCL3-CCL4 chemokine gene cluster has been shown to influence HIV-1 transmission and disease progression^21,22^. Here, we report that plasma levels of CCL18 are significantly increased during HIV-1 infection and negatively correlate with CD3^+^CD4^+^ T cell counts in patients on combination antiretroviral therapy (cART) with a suppressed viral load.

## Results

### The Levels of Plasma CCL18 Are Significantly Increased During HIV-1 Infection

**Figure 1:**
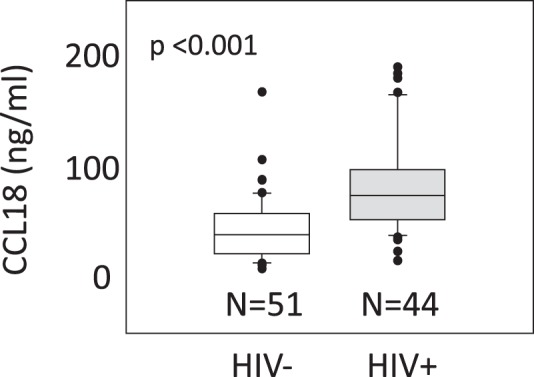
Plasma levels of the TH2 chemokine CCL18 are significantly increased in HIV-1 infected patients. CCL18 levels were analyzed by ELISA in plasma samples collected from the blood of 44 HIV-1 infected patients (irrespective of treatment status) and 51 control individuals (cohort 1). P value was calculated by a t-test.

Numerous studies have shown imbalanced TH1/TH2 cytokine responses during HIV-1 infection and have suggested their involvement in disease pathogenesis. However, the role of TH2 chemokines in HIV-1 pathogenesis is not well defined. We, therefore, investigated the expression of TH2 chemokines during HIV-1 infection. We focused on the TH2 chemokine CCL18, which is a marker of M2 macrophages and has been previously linked to several TH2 related pathological conditions^21^. The expression levels of CCL18 were analyzed in plasma samples collected from 44 HIV-1-infected patients and compared to the levels detected in the plasma of 51 age-matched healthy individuals from cohort 1 as mentioned in the method section. The data showed that CCL18 levels were significantly increased in plasma of HIV-1-infected patients as compared to uninfected controls (Fig. 1). The mean plasma CCL18 level of healthy individuals was 44 ng/ml, ranging from 7.9 to 167 ng/ml. The mean plasma CCL18 level of HIV-1 infected individuals was 89 ng/ml, ranging from 24 to 216 ng/ml. The range of detected CCL18 levels in both groups showed a relatively high variation. An unpaired t-test confirmed the fact that the difference between the two groups of CCL18 levels was significantly different from each other (P < 0.001). The normality assumption required for the unpaired t-test is well satisfied with the large enough sample sizes available in each group (>30).

### Correlation Between CCL18 Levels and Viral Load in the Group of Untreated HIV-1-Infected Patients

**Figure 2:**
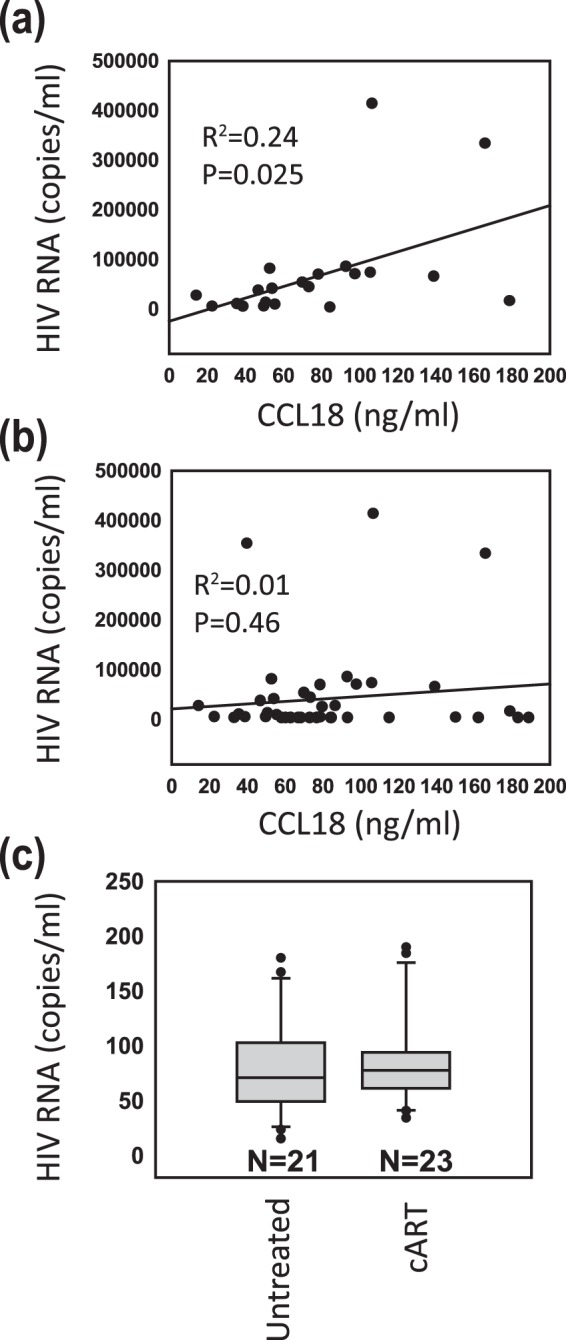
Plasma levels of CCL18 correlate with viral load in the group of untreated HIV-1-infected patients. (**a**) Regression analysis of the correlation between plasma CCL18 levels detected in the group of untreated HIV-1 infected patients and HIV-1 mRNA copies detected in plasma isolated from the same patients (**b**) Regression analysis of the correlation between CCL18 levels detected in all HIV-1 infected patients (regardless of treatment status) and HIV-1 mRNA copies detected in plasma isolated from the same patients. (**c**) CCL18 levels detected in plasma isolated from 21 untreated and 23 cART-treated HIV-1-infected patients. All samples are from cohort 1. P value was calculated by a t-test.

Plasma CCL18 levels from each patient were then plotted against the number of HIV-1 mRNA copies detected in the same plasma sample and analyzed. Regression analysis showed a significant correlation between CCL18 levels and viral load in the plasma of untreated HIV-1-infected patients (Fig. 2a) suggesting an interplay between CCL18 and the virus. However, when we analyzed samples from all 44 patients, regardless of their treatment status, no correlation between CCL18 levels and viral load was detected (Fig. 2b). Figure 2b also depicts a noticeable lower plasma viral load for this group compared to the untreated patients. In both cases, the normality assumption required for the use of unpaired t-tests for the analysis was well satisfied with the large sample size (>30) we had. When we compared plasma CCL18 expression in HIV-1-infected patients on cART to those detected in plasma collected from untreated HIV-1-infected patients using an unpaired t-test, no significant difference was detected (Fig. 2c). This data suggests that increased CCL18 plasma levels persist even after the suppression of the viral load by cART. Altogether this data suggest that suppression of viral load was not sufficient enough to normalize previously increased CCL18 levels. Shapiro-Wilk test was used to check the Normality assumption among the CCL18 levels of HIV-1 infected untreated patients and CCL18 levels of HIV-1 infected cART-treated patients. The normality assumption required for the t-test was satisfied. The corresponding Shapiro-Wilk test’s p-values for the CCL18 values of the HIV-1 infected untreated patients and HIV-1 infected cART-treated patients are 0. 72 and 0.84 respectively.

### Plasma CCL18 Levels Negatively Correlate with CD4+ T Cell Counts in HIV-1-infected Patients on cART with Suppressed Viral Load

**Figure 3:**
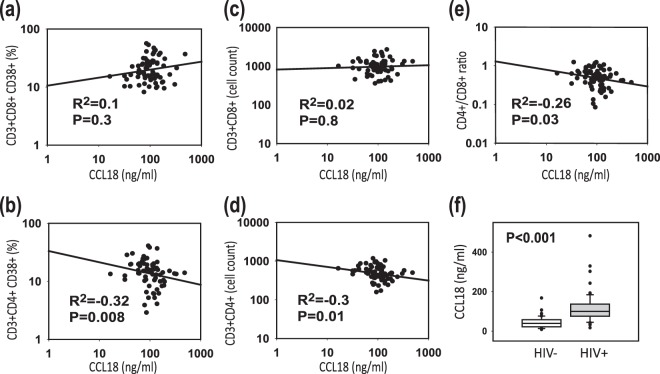
Plasma levels of CCL18 negatively correlate with CD4+ T cell counts in HIV-1 infected patients. CCL18 levels were analyzed by ELISA in plasma samples collected from 68 HIV-1 infected patients on cART with undetectable plasma viral load (cohort 2). The CD3+CD4+ and CD3+CD8+ cell counts and the expression of CD38 on both cell populations were determined by flow cytometry. Correlation of CCL18 levels with (**a**) CD3+CD8+CD38+ cells, (**b**) CD3+CD4+ CD38+ cells, (**c**) CD3+CD8+ cell count, (**d**) CD3+CD4+ cell count, and (**e**) CD4/CD8 ratio was determined by Spearman Correlation Coefficients using SAS software. (**f**) Cohort 2 CCL18 levels detected in the plasma of 68 HIV-1-infected patients on cART with undetectable plasma viral load as compared to control individuals.

Treatment of HIV-1-infected patients with cART generally results in decreased plasma viral load and an increase in the CD4+ T cells. However, immune dysfunctions such as chronic immune activation accompanied by low CD4+ T cell counts persist in a subset of patients receiving cART^23^. In light of these findings, we sought to determine whether there was the persistence of high CCL18 levels associated with immune abnormalities detected in patients on cART. We analyzed plasma CCL18 levels collected from 68 HIV-1-infected patients undergoing cART, all of whom had suppressed plasma viremia with a viral load below 50 copies per ml. These patients were enrolled in the 2^nd^ cohort described in the material and method section. The Plasma CCL18 levels of these patients were compared to the expression of immune activation marker CD38 on CD8+ and CD4+ T cells and to total CD4+ and CD8+ T cell counts. The expression of immune activation marker CD38 in CD8+ T cells increased along with CCL18 levels, however, a statistically significant correlation was not found (Fig. 3a). Contrary to CD8+ T cells, a significant negative correlation between CCL18 levels and CD4+ CD38+ T cells was detected (Fig. 3b). Importantly, while no correlation between CCL18 and CD8+ T cell counts was detected (Fig. 3c) we observed a significant negative correlation between CCL18 and CD4+ T cell counts (Fig. 3d). The data further reflected a negative correlation between the CD4+ /CD8+ ratio and CCL18 levels (Fig. 3e). Figure 3f shows that even though patients in this cohort (cohort 2) had all undetectable viral load their plasma CCL18 levels were increased as compared to controls. An association between high CCL18 levels and low CD4+ T cells in patients with undetectable viral load raises the possibility that CCL18 and/or CCL18-producing cells may interfere with CD4+ T cell reconstitution in HIV-1-infected patients on cART. The normality assumption required for the use of unpaired t-tests for this analysis was well satisfied with the large sample size (>30) we had.

### Plasma CCL17 and CCL22 are Increased in HIV-1 Patients on cART but do not Correlate with CD4+ T Cell Counts

**Figure 4:**
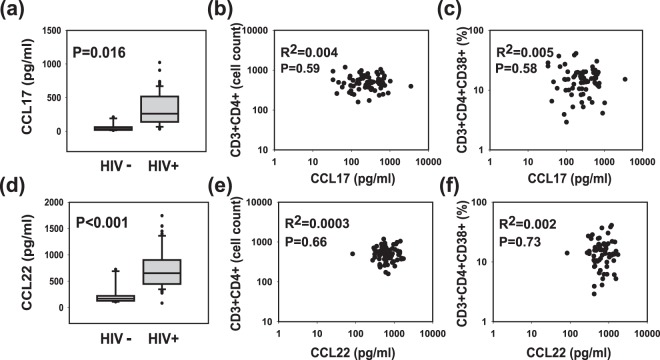
Plasma levels of the Th2 chemokines CCL17 and CCL22 in HIV-1 infected patients. CCL17 and CCL22 were analyzed by ELISA in plasma collected from 68 HIV-1-infected patients on cART with undetectable plasma viral load and compared to those detected in healthy controls. Upper panels show plasma levels of CCL17 (**a**), and correlation of plasma CCL17 levels with CD3+CD4+ (**b**) and CD3+CD4+ CD38+ (**c**) positive cells. Lower panels show plasma levels of CCL22 (**d**), and their correlation with CD3+CD4+ (**e**) and CD3+CD4+ CD38+ (**f**) positive cells. All samples are from cohort 2. P values were calculated using t-test.

CCL17 and CCL22 are two members of the Beta-chemokine family with TH2 associated expression pattern^22,23^. We used the same plasma samples collected from 68 HIV-1-infected patients on cART (cohort 2) to investigate CCL17 and CCL22 expression to determine whether low CD4+ T cell counts are associated with a general increase in the expression of TH2 chemokines. Similar to CCL18, we detected increased plasma levels of CCL17 (Fig. 4a) in HIV-1-infected patients undergoing cART. However, no correlation between CCL17 and CD4+ T cell counts (Fig. 4b) or percent of CD4+ CD38+ T cells (Fig. 4c) was detected. Analysis of CCL22 yielded similar data showing an increase in plasma CCL22 (Fig. 4d). However, as with CCL17, no correlation with CD4+ T cell counts (Fig. 4e) or CD4+ CD38+ cells was detected (Fig. 4f). Taken together, these data suggest that although all three TH2 chemokines are increased during HIV-1 infection, only CCL18 and/or CCL18-producing cells play a role in CD4+ T cell recovery as experienced by some HIV-1-infected patients undergoing cART. The normality assumption required for the use of unpaired t-tests for this analysis was well satisfied with the large sample size (>30) we had.

### *In Vitro* Analysis of the Effect of CCL18 on CD4+ T Cells

**Figure 5:**
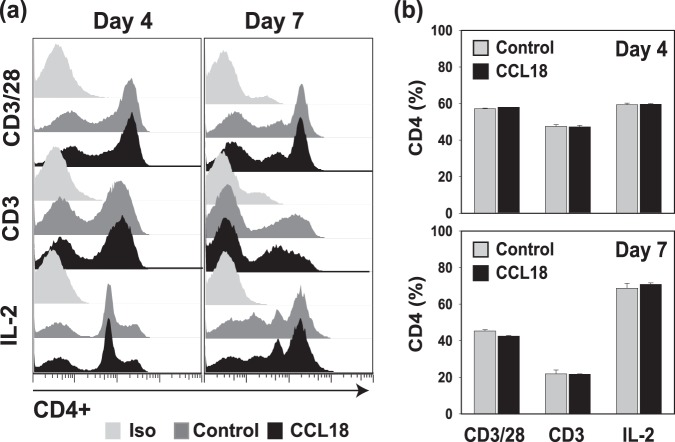
*In vitro* analysis of the CD4+ T cell population cultivated in the presence of CCL18. (**a**) Representative staining of CD4+ lymphocytes cultivated with human recombinant CCL18 (500 ng/ml) and activated with CD3/CD28, CD3, or IL-2. Control shows staining of activated lymphocytes cultivated without CCL18. In parallel, cells were stained with mouse isotype antibody (Iso) to rule out non-specific binding. (**b**) Percent of CD4+ T cells in lymphocytes cultivated with or without CCL18 as described above. Data show results of one representative experiment out of three, each performed in duplicate.

Based on previous observations suggesting an effect of chemokines on the expression of immune cells, we sought to determine whether CCL18 might affect CD4+ T cell counts directly by interfering with CD4 detection^24,25^. To address this question we analyzed CD4 expression on primary lymphocytes cultivated *in vitro* in the presence of human recombinant CCL18 and activated with CD3/CD28 or CD3 alone. Obtained results did not show a decrease in CD4+ T cells in CCL18-treated cultures with respect to untreated controls (Fig. 5a). Moreover, the percentage of CD4 expressing lymphocytes were similar in CCL18-treated and control cells regardless of the mode of activation (Fig. 5b) suggesting that the negative correlation between CCL18 levels and CD4+ T cell counts may not be the result of a direct effect of CCL18 on CD4 expression.

## Discussion

The present study demonstrates that CCL18 levels are significantly increased in HIV-1-infected patients and correlate with viral load in the group of untreated patients. The elevation in CCL18 levels persists even during cART suggesting that the suppression of viral replication may not be sufficient enough to normalize CCL18 production. This further suggests that although HIV-1 may be the initial trigger leading to increased expression of CCL18, other mechanisms such as a continuing TH1/TH2 imbalance^8^, dysregulation in the expression and function of co-stimulatory molecules^26–28^, or other mechanisms that contribute to chronic immune activation might be involved in enhanced CCL18 production during cART. Furthermore, this observation presents a possible scenario in which the initial induction of CCL18 expression might be augmented via the self-amplification capability of CCL18 (our unpublished results). These results also suggest that the mechanisms regulating CCL18 production during HIV-1infection are distinct from those found in Gaucher disease, where Gaucher cells (glycolipid-laden macrophages) are the prominent source of CCL18 and production of CCL18 decreases during therapy^29^.

In breast cancer patients, tumor-associated macrophages (TAMs) with M2 phenotype abundantly produce CCL18, and its expression in blood or cancer stroma is associated with metastasis and reduced patient survival^30,31^. Based on this data we can speculate that increased CCL18 levels represent a marker of M2 macrophages, which might suppress CD4+ T cell proliferation and/or interfere with CD4+ T cell recovery in cART-treated patients with a suppressed viral load. One study found that cART has a limited effect on reducing the polarization of CD4+ T cells to the TH2 subset^32^. Currently, HIV-1 treatment strategies are in development that aims to disrupt macrophage polarization by inhibiting certain molecular signals that underlie their different activation states^33^.

To the best of our knowledge, this is the first study to indicate the possibility that increased production of CCL18 may contribute to immune dysfunctions detected in HIV-infected patients. Our data show a correlation between increased CCL18 levels and decreased CD4 counts, particularly in the population of activated CD4 cells expressing CD38 activation marker, in patients with the suppressed viral load. While the correlation is significant it is not as impressive as we expected. We assume that analysis of a larger cohort of patients may yield stronger correlation. We further recognize that our study lack complete patients’ demographic data, particularly the proportion of male and female participants. Although recent study analyzing CCL18 levels in COPD patients detected similar levels in man and women without COPD^34^ it will be useful to analyze larger cohort with more detailed patient demographic data.

It has been shown previously that HIV-1 infection of macrophages activates resting T lymphocytes, rendering them susceptible to HIV-1 infection^33^. This activation is mediated via HIV-1 Nef, which promotes the release of soluble CD23 and soluble ICAM, which in turn, up-regulates co-stimulatory receptors on B cells that interact with corresponding ligands on T cells, thus rendering them permissive to HIV-1 infection^35,36^. By attracting naïve T cells, B cells, and differentiated macrophages, CCL18 can increase the pool of HIV-1 targets at the site of CCL18 production and HIV-1 replication. Further studies are needed to determine whether CCL18 or CCL18-producing M2 macrophages play a role in CD4+ T cell dysfunctions detected in HIV-1-infected patients on cART.

## Methods

### Patients and Sample collection

Ninety-five subjects, including 44 HIV-1-infected individuals and 51 age-matched healthy controls were enrolled at Northwell Health. Of the 44 HIV-1-infected subjects, 23 were undergoing cART and 21 were not on treatment at the time of plasma collection (cohort 1). In addition, 68 HIV-1-infected patients on cART all with suppressed plasma viremia (viral load <50 copies/ml) were enrolled at the Miami VA Medical Center (cohort 2). Plasma HIV-1 RNA copy levels were evaluated using Plasma HIV-1 RNA quantification by NucliSense HIV-1 QT assay (Organon) as per manufacturer’s instruction. The Northwell Health IRB (LIJ IRB No: 94136 and LIJ IRB exemption No 06–016) as well as Miami VA Medical Center IRB (IRB No 3499.01) approved the study and entire study was performed according to both institutional guidelines and regulatory standards set by the National Institutional Health (NIH). The age of enrolled patients ranged from 20 to 64 years. Each patient had  given informed consent for participation in the study.

### Cells

Peripheral blood mononuclear cells from healthy donors were separated on a Ficoll-Hypaque gradient. Monocytes were eliminated by plastic adherence. Lymphocytes were resuspended in RPMI 1640 medium supplemented with 10% heat-inactivated fetal calf serum (BioWhittaker) and stimulated either with anti-CD3/CD28, anti-CD3 alone (both from Thermo Fisher Scientific, USA), or human recombinant IL-2 at the final concentration of 20 U/ml (equal to 10 ng/ml; Roche, USA). The anti-CD3 antibody was pre-coated to plates (1 µg/ml). Anti-CD28 (1 µg/ml) was added in soluble form. The anti-CD3 and anti-CD-28 antibodies were purchased from Beckman-Coulter, USA.

### ELISA

Plasma CCL18, CCL17, and CCL22 levels were analyzed by commercially available ELISA (R&D Systems) according to the manufacturer’s instruction.

### Flow Cytometry

Patient samples: The expression of the immune activation marker CD38 on CD4 lymphocytes (CD3^+^CD4^+^) and CD8 lymphocytes (CD3+CD8+) and lymphocyte (CD3^+^CD4^+^ and CD3+CD8+) counts in HIV-1-infected patients were determined by whole-blood direct immunofluorescence staining. For each sample, 100 µl of whole blood was incubated for 15 minutes at room temperature with 10 μl of fluorochrome-conjugated anti-CD3, anti-CD4 or anti-CD8, and anti-CD38 antibodies, or appropriate fluorochrome-conjugated isotype control antibodies (Beckman Coulter, CA, USA). After incubation, the samples were lysed and fixed with Q-Prep (Beckman Coulter, CA, USA) and analyzed on an Epics XL-MCL flow cytometer (Beckman Coulter, CA, USA). Absolute numbers of CD3+CD4+ and CD3+CD8+ lymphocytes were determined by multiplying the total peripheral lymphocyte counts by percent of positive cells for each marker.

*In vitro* staining: the CD4+ expression on lymphocytes cultivated with or without human recombinant CCL18 (500 ng/ml; PeproTech USA) and activated with anti-CD3/CD28, anti-CD3, or IL-2 as described above was determined by flow cytometry. On day 4 and day 7 following CCL18 treatment, cells were stained with fluorochrome-labeled anti-CD3 and anti-CD4 antibodies (10 μl per sample; Beckman Coulter, CA, USA). The CD4+ cells were analyzed on CD3+ gated cell population using FlowJo data analysis software.

### Statistical analysis

Statistical analysis was performed by SigmaPlot 11.0 software. Data were compared using the unpaired t-tests. P-values below 0.05 were considered significant. The normality assumption required for the unpaired t-tests was well justified with the large sample size, except for one case. In that case, where we test for CCL18 level differences between the HIV-1 infected untreated patients and HIV-1 infected cART-treated patients, the assumption of normality required for the unpaired t-test was confirmed by the Shapiro-Wilk normality test (Fig. 2c). Correlation and curve fit analyses were performed by Spearman Correlation using SAS software.
